# Estimation of the Effective Dose of Phenylephrine Infusion for Preventing Spinal Anesthesia-Induced Hypotension in Elective Cesarean Deliveries: A Randomized Controlled Trial

**DOI:** 10.7759/cureus.96873

**Published:** 2025-11-14

**Authors:** Soumya R Sahoo, Devdutta Raiguru, Anusha Kakarla, Saswati Das

**Affiliations:** 1 Department of Anesthesiology, Kalinga Institute of Medical Sciences, Bhubaneswar, IND; 2 Department of Anesthesiology, Institute of Medical Sciences (IMS) and Sum Hospital Campus II, Bhubaneswar, IND; 3 Department of Neuroanesthesiology, All India Institute of Medical Sciences, New Delhi, IND

**Keywords:** caesarean section, fetal acidosis, mean arterial pressure, spinal anesthesia, vasopressor

## Abstract

Introduction: Spinal anesthesia for cesarean delivery may be associated with hypotension and fetal acidosis. Phenylephrine, an α1 adrenergic receptor agonist, is effective in treating hypotension with the benefit of less placental transfer.

Objective: The objective of this study was to determine the optimum dose of prophylactic phenylephrine infusion for preventing spinal anesthesia-induced hypotension in women undergoing elective cesarean delivery and to assess its effect on fetal outcomes, as measured by APGAR scores at 1, 5, and 10 minutes.

Methodology: Two hundred and forty patients were included in this double-blinded study. Women undergoing elective cesarean section were preloaded with crystalloids and then randomly allocated to receive prophylactic Phenylephrine infusion at either 25, 50, 75, or 100 µg/minute immediately after spinal anesthesia (Groups A, B, C, and D, respectively). Maternal hemodynamic parameters, total dose of phenylephrine used, APGAR score, and incidence of maternal complications like reactive hypertension, bradycardia, nausea, and vomiting, as well as fetal complications like fetal acidosis, were noted. The study was registered with the Clinical Trials Registry of India (CTRI/2019/07/020231, dated July 17, 2019)

Results: Demographic parameters were comparable across all four groups (*P* > 0.05). The incidence of maternal hypotension was lowest in Group B (50 µg/minute) at 15.0%, compared to 41.7% in Group A, 30.0% in Group C, and 23.3% in Group D (*P* = 0.001). Maternal adverse events such as nausea/vomiting occurred in 5% of Group B, 28.3% of Group A, 31.7% of Group C, and 20% of Group D (*P* = 0.016). Mean phenylephrine requirements were 692 ± 67 µg (Group A), 1,269 ± 120 µg (Group B), 592 ± 115 µg (Group C), and 439 ± 83 µg (Group D) (*P* < 0.001). Mean APGAR scores at 1 minute were above 8.4 for all groups; all neonates had mean APGAR scores above 9.8 by 5 minutes (*P* > 0.05). Maternal complications such as bradycardia ranged from 25% to 63% across groups. The frequency of bradycardia was significantly higher in Groups C (63.3%) and D (60%) than in Groups A (26.7%) and B (25%) (*P* < 0.001). The 95% confidence intervals for major outcomes indicated statistical significance for these comparisons.

Conclusions: We concluded that starting prophylactic Phenylephrine infusion at 50 µg/minute immediately after spinal anesthesia was an effective and simple method of reducing the incidence, frequency, and magnitude of hypotension in elective cesarean section with no adverse effect on neonatal outcome.

## Introduction

Spinal anesthesia has a time-honored place among the options of anesthesia for cesarean section. The advantages of spinal anesthesia include the relative ease of administration, rapid onset, and provision of ideal surgical conditions, while also avoiding possible neonatal cardiovascular and respiratory depression, and minimizing risks associated with airway management [1,2].

Recent advances in obstetric anesthesia have further highlighted the importance of individualized strategies to minimize maternal hypotension and improve neonatal outcomes during cesarean section. In particular, the implementation of prophylactic vasopressor infusions, such as phenylephrine, has been shown in multiple recent studies and meta-analyses to more effectively prevent maternal hypotension compared to reactive bolusing, with reported reductions in the incidence of hypotensive episodes and associated side effects such as nausea and vomiting. Additionally, contemporary guidelines emphasize a multimodal approach that includes judicious use of intravenous fluids (preferably co-loading), physical methods (such as leg elevation), and pharmacological strategies for optimal hemodynamic control. The refinement of spinal anesthesia techniques and vasopressor dosing regimens continues to evolve, aiming to further minimize maternal complications while consistently yielding favorable neonatal outcomes, thereby supporting proactive, standardized protocols in elective cesarean delivery [3-5].

0.5% Hyperbaric bupivacaine is the most common intrathecal local anesthetic used for cesarean section in our country. However, it causes hypotension as a part of its pharmacological effects. For adequate surgical analgesia, a high block to at least T6 level is necessary, but the concomitant vasodilatation thus produced predisposes to hypotension in the mother, even if we avoid aorto-caval compression. Hypotension following spinal anesthesia is a common complication that is further aggravated in pregnant patients as systemic vascular resistance decreases by 20% during the third trimester of pregnancy [6,7]. Post-spinal hypotension is reported in 50%-90% of parturients with a decrease in utero-placental blood flow and significant fetal acidosis [8,9]. The usual approach is reactive rather than proactive, i.e., hypotension is allowed to develop and then treated with intravenous fluids and boluses of vasopressors. Phenylephrine, an α1 adrenergic receptor agonist, is not only effective in reducing hypotension but also has less placental transfer to the fetus and hence causes less fetal acidosis than Ephedrine [10-12]. However, the ideal dosing regimen for Phenylephrine infusion is not yet standardized [13]. In this study, prophylactic infusion of four different doses of Phenylephrine started immediately after the induction of spinal anesthesia was investigated.

The primary outcome was to obtain an optimum dose of Phenylephrine infusion for preventing hypotension due to spinal anesthesia in women undergoing elective cesarean delivery. The secondary outcome was to prevent fetal hypoxia and acidosis following maternal hypotension due to spinal anesthesia, as assessed by APGAR score at 1, 5, and 10 minutes.

## Materials and methods

This was a double-blinded, randomized control study conducted between February 2020 to January 2022 on American Society of Anesthesiologists (ASA) grade II pregnant females, aged>18 years, with gestational age ≥ 37 weeks of pregnancy and planned for elective cesarean section under spinal anesthesia.

The study was conducted at the Department of Anesthesiology, Kalinga Institute of Medical Sciences, after obtaining permission from the institutional ethics committee (KIMS/KIIT/IEC/115/2018) and informed written consent from all participants. The study was registered with CTRI/2019/07/020231.

Inclusion and exclusion criteria

All females with a gestational age of more than 37 weeks and posted for elective cesarean delivery were included in this study. This included patients with well-controlled comorbidities (type 2 diabetes mellitus, gestational diabetes, hypertension, and hypothyroidism).

Patients belonging to ASA grades III and IV, or those with any uncontrolled systemic disorders such as diabetes mellitus, hypertension, preeclampsia, eclampsia, or HELLP (Hemolysis, Elevated Liver enzymes, and Low Platelet count) syndrome were excluded. Individuals with a history of cardiovascular disease, seizure disorders, blood dyscrasias, hepatic or renal impairment, or severe airway or respiratory disease were also not included. Patients with known hypersensitivity or allergy to phenylephrine, bupivacaine, or atropine were excluded, as were those with spinal deformities, infection at the site of intended spinal anesthesia, or any contraindication to spinal block. Pregnant women exhibiting fetal distress or known fetal abnormalities were also excluded. Finally, patients unwilling to participate in the study or provide written informed consent were not considered eligible for inclusion.

Sample size calculation

**Figure 1:**
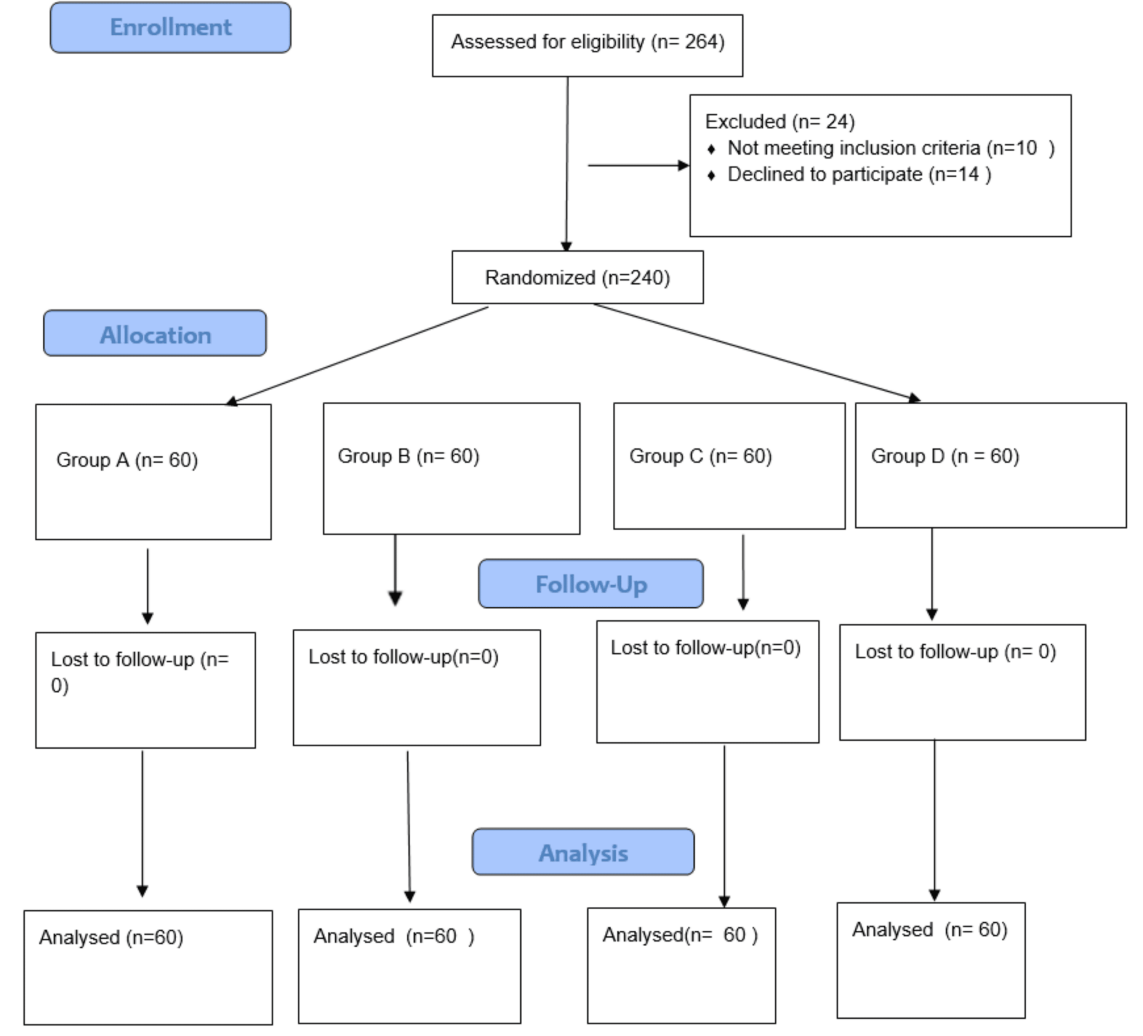
CONSORT diagram. A total of 264 participants were assessed for eligibility. Of these, 24 were excluded (14 declined consent, 10 did not meet the inclusion criteria). The remaining 240 were randomized (60 each in four groups). None were lost to follow-up, and 60 in each group were analyzed. CONSORT, Consolidated Standards of Reporting Trials

The sample size was calculated based on previous studies comparing vasopressor infusions for the prevention of spinal anesthesia-induced hypotension in elective cesarean sections. Considering an effect size of 0.45 between groups, with a two-sided significance level of 5%, study power of 90%, and a 95% confidence interval, the minimum required sample size was found to be 78 patients per group. To account for practical considerations and ensure adequate statistical validity, each group in our trial comprised 60 subjects, providing a total sample size of 240 parturients. This sample size was considered sufficient to detect clinically meaningful differences in maternal hemodynamics and neonatal outcomes among groups receiving different prophylactic phenylephrine infusion rates. Based on a computerized randomization technique, these subjects were randomly allocated to the four study groups (Figure 1).

All participants were prepared for elective cesarean delivery with nil per oral for 8 hours before surgery and all pre-medication as per hospital protocol. All participants were pre-loaded with 10 mL/kg of Ringer's lactate (RL) 15 minutes before subarachnoid block (SAB) after securing intravenous access with an 18-gauge cannula, and then continued with 10 mL/kg/hour during the surgery. Under all aseptic precautions, an SAB was given with a 25-gauge Quincke’s needle at L3-L4 or L4-5 space using 2.4 mL of 0.5% hyperbaric bupivacaine in the sitting position. After administration of an SAB, patients were placed supine with left uterine displacement. Evaluation of sensory, autonomic, and motor blockade was done before surgery. A sensory level of T6 was achieved before commencing the surgery. If the spinal block failed, the patient was given general anesthesia as per institutional protocol and considered a dropout from the study. Maternal HR, systolic BP, diastolic BP, and mean arterial pressure (MAP) were noted before preloading, after preloading, before giving SAB, immediately after SAB, every one-minute interval till the baby was delivered, and thereafter, at every three-minute interval till the end of surgery. The patients were randomly allocated into four groups: A, B, C, and D. Immediately after SAB, Groups A, B, C, and D were started with phenylephrine infusion at the rate of 25, 50, 75, and 100 µg/minute respectively, all diluted to 50 mL with normal saline, with an infusion syringe pump at 1 mL/minute. If MAP was ≥ 20% above baseline value or HR fell below 60 bpm, infusion was stopped. Our protocol was unique in that once an infusion was stopped (either for bradycardia or hypertension), it was not restarted. If hypotension persisted, defined by MAP ≤ 20% of baseline or MAP ≤ 65 mmHg, whichever was higher, a top-up dose of 1 mL of 10 µg/mL of phenylephrine was administered. In case of bradycardia (HR ≤ 50 bpm), atropine 0.6 mg was given.

Statistical analysis

The data on categorical variables were shown as *n* (% of cases), and the data on continuous variables were presented as mean and standard deviation (SD) or standard error of mean (SEM) across four study groups. The inter-group statistical comparison of continuous variables was done using analysis of variance (ANOVA) with Bonferroni’s correction for multiple group comparisons. The inter-group statistical comparison of the distribution of categorical variables was tested using the chi-square test with Bonferroni’s correction for multiple group comparisons. The underlying normality assumption was tested before subjecting each variable to analysis of variance (ANOVA). In the entire study, the *P*-values less than 0.05 were statistically significant. All the hypotheses were formulated using two-tailed alternatives against each null hypothesis (hypothesis of no difference). The entire data was statistically analyzed using SPSS version 22.0 (IBM Corp., Armonk, NY) for MS Windows. *P*-values by ANOVA with Bonferroni’s post hoc test for multiple group comparisons. *P*-value < 0.05 was statistically significant.

## Results

A total of 240 ASA grade II parturients were enrolled and evenly distributed into four groups (A, B, C, and D) according to the prophylactic phenylephrine infusion rates used. All groups were comparable with respect to demographic and baseline clinical variables.

Baseline characteristics

The mean age, gestational age, weight, hemoglobin, and amniotic fluid index (AFI) did not show any statistically significant difference among groups (*P* > 0.05). The incidence of singleton pregnancies and maternal comorbidities such as diabetes or hypothyroidism was comparable across all groups (*P* = 0.999). These similarities confirmed that the study groups were well matched at baseline (Table 1).

**Table 1:** Baseline characteristics of study group. AFI, amniotic fluid index; Hb, hemoglobin; SD, standard deviation

Parameter	Group A (*n *= 60)	Group B (*n *= 60)	Group C (*n *= 60)	Group D (*n *= 60)	*P*-value
Age (years, mean ± SD)	29.68 ± 5.07	29.10 ± 4.12	28.50 ± 4.53	28.18 ± 4.42	0.999
Gestational age (weeks)	38.05 ± 1.05	37.93 ± 1.10	37.82 ± 1.03	37.90 ± 1.05	0.999
Weight (kg)	68.68 ± 10.14	68.50 ± 11.83	69.27 ± 11.33	66.20 ± 10.72	0.999
AFI (cm)	10.98 ± 4.24	12.56 ± 4.00	12.73 ± 4.53	12.58 ± 4.25	0.266
Hb (g/dL)	11.43 ± 1.24	11.48 ± 1.12	11.86 ± 1.07	11.49 ± 1.06	0.235
Singleton pregnancy (%)	91.7	98.3	91.7	98.3	0.564
Maternal comorbidity (%)	33.3	30	36.7	45	0.999

Baseline hemodynamic parameters

Baseline maternal heart rate, systolic blood pressure, diastolic blood pressure, and MAP were not significantly different between groups (*P* > 0.05). This indicated uniform hemodynamic conditions before initiation of spinal anesthesia and phenylephrine infusion (Table 2).

**Table 2:** Baseline characteristics. HR, heart rate; SBP, systolic blood pressure; DBP, diastolic blood pressure; mmHg, millimeters of mercury; bpm, beats per minute

Parameter	Group A	Group B	Group C	Group D	p Value
HR (bpm)	83.77 ± 10.00	86.22 ± 10.27	87.28 ± 9.94	86.75 ± 8.79	0.574
SBP (mmHg)	118.30 ± 11.26	117.50 ± 8.96	120.90 ± 8.86	117.00 ± 9.62	0.866
DBP (mmHg)	77.43 ± 8.46	74.53 ± 7.42	77.12 ± 8.11	76.07 ± 7.55	0.447
MAP (mmHg)	90.40 ± 8.89	88.23 ± 7.37	91.15 ± 7.83	89.25 ± 7.44	0.809

Infusion and hemodynamic outcomes

**Figure 2:**
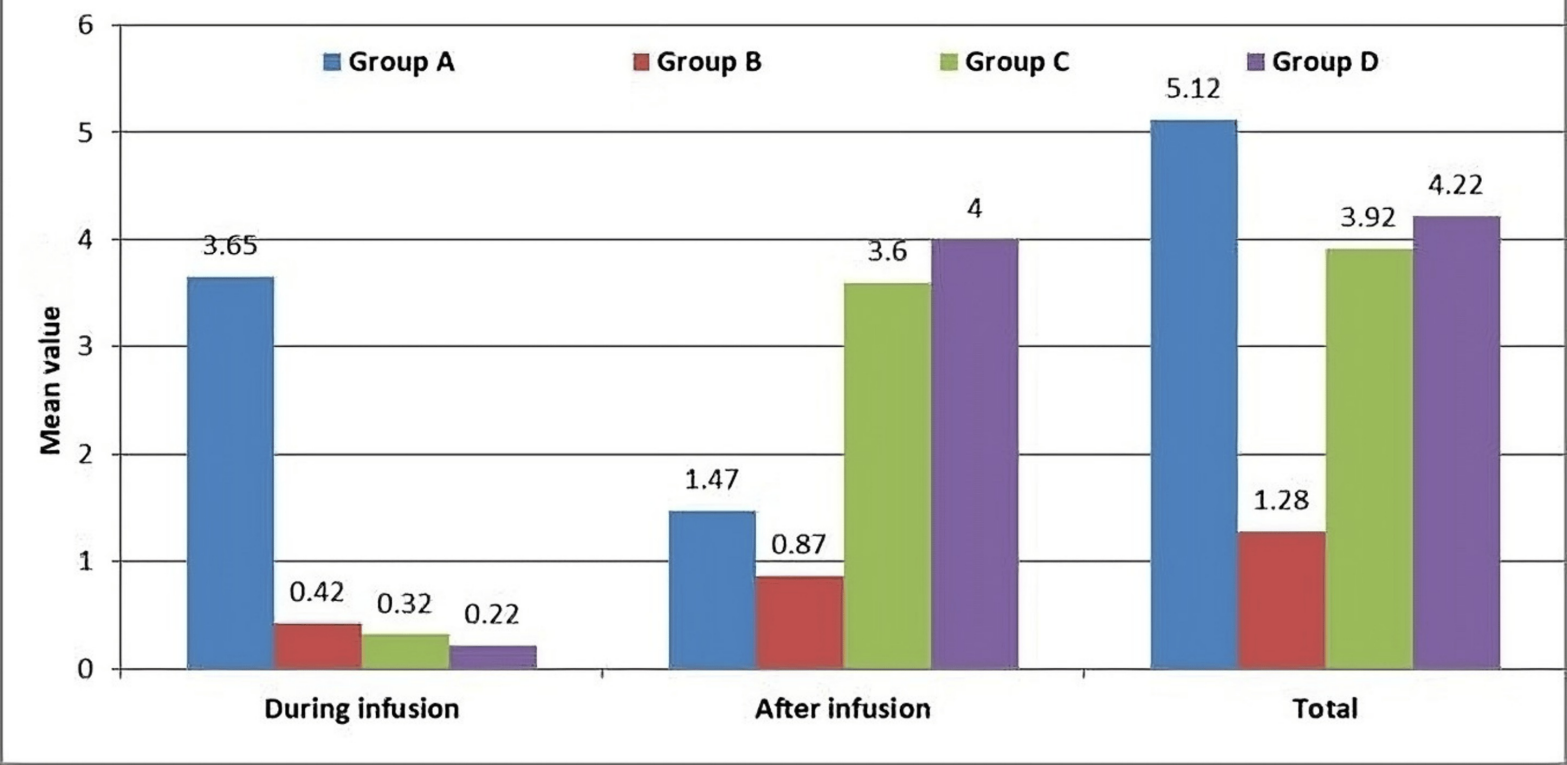
Inter-group distribution of mean number of phenylephrine boluses. Groups A, B, C, and D correspond to prophylactic phenylephrine infusion rates of 25, 50, 75, and 100 mcg/minute, respectively, administered after spinal anesthesia for elective cesarean delivery. The graph illustrates the mean (± SD) number of phenylephrine bolus doses required during the infusion period in each group. Group B required significantly fewer boluses compared to the other groups *P* < 0.001). Statistical analysis was performed using analysis of variance (ANOVA) with Bonferroni correction.

The mean infusion stop time differed significantly across groups (*P* = 0.001). Groups A and B maintained infusion longer (approximately 25 minutes) without complications, whereas Groups C and D showed early discontinuation (7.38 ± 1.54 minutes and 3.97 ± 0.84 minutes, respectively) due to bradycardia or reactive hypertension. The mean number of rescue boluses required during infusion was significantly higher in Group A compared to Groups B, C, and D (*P* = 0.001) (Figure 2). Similarly, post-infusion bolus requirements were greater in Groups C and D (*P* = 0.001). Group B required the least total number of phenylephrine boluses overall and maintained stable blood pressure with minimal side effects.

**Figure 3:**
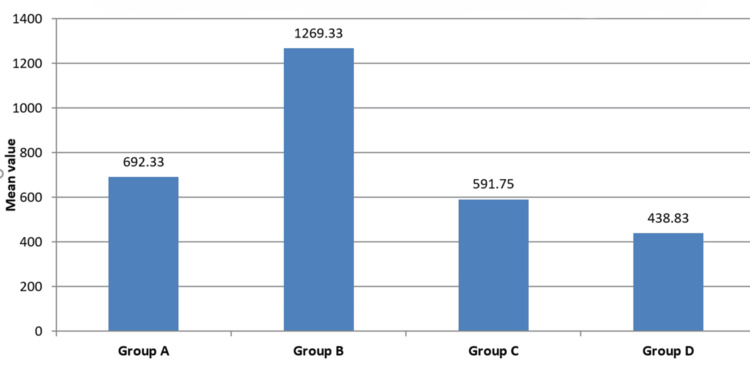
Inter-group distribution of mean total phenylephrine used. Groups A, B, C, and D represent prophylactic phenylephrine infusion rates of 25, 50, 75, and 100 mcg/minute, respectively, following spinal anesthesia. The graph shows the mean total phenylephrine dose (micrograms, ± SD) administered per group. Group B required a significantly higher total dose compared to other groups (*P* < 0.001). Statistical significance was determined using analysis of variance (ANOVA) with Bonferroni correction.

The total amount of phenylephrine used varied significantly between groups (*P* = 0.001), with Group B requiring moderate cumulative doses (1269.33 ± 119.52 µg) compared to lower doses in Groups C and D and comparatively less in Group A (Figure 3).

The results revealed significant inter-group differences in phenylephrine administration and infusion stop times (*P* = 0.001). The mean infusion stop time was comparable for Groups A (25.00 minutes, 95% CI: 20.23-29.77) and B (25.17 minutes, 95% CI: 20.46-29.88), but markedly shorter in Group C (7.38 minutes, 95% CI: 4.36-10.40) and D (3.97 minutes, 95% CI: 2.32-5.62).​

During the infusion period, the mean number of phenylephrine boluses required was highest in Group A (3.65, 95% CI: 2.61-4.69), while Groups B, C, and D needed substantially fewer boluses (0.42 (95% CI: 0.05-0.79), 0.32 (95% CI: 0.14-0.50), and 0.22 (95% CI: 0.08-0.36), respectively; *P* = 0.001). After discontinuation of the infusion, Groups C and D required more bolus doses (3.60 (95% CI: 2.81-4.39) and 4.00 (95% CI: 3.04-4.96)), compared to Groups A (1.47, 95% CI: 0.76-2.18) and B (0.87, 95% CI: 0.22-1.52; *P* = 0.001).​

Total phenylephrine consumption was highest in Group B (1269.33 µg, 95% CI: 1035.97-1502.69), followed by Group A (692.33 µg, 95% CI: 561.64-823.02), and was lowest for Groups C (591.75 µg, 95% CI: 365.90-817.60) and D (438.83 µg, 95% CI: 276.94-600.72; *P* = 0.001) (Table 3).

**Table 3:** Infusion and hemodynamic outcomes. mcg, microgram; SEM, standard errors of mean; CI, confidence interval

Parameter	Group A	Group B	Group C	Group D	95% CI Group A	95% CI Group B	95% CI Group C	95% CI Group D	*P*-value
Infusion stop time (minutes ± SEM)	25.00 ± 2.43	25.17 ± 2.40	7.38 ± 1.54	3.97 ± 0.84	20.23-29.77	20.46–29.88	4.36-10.40	2.32-5.62	0.001
Phenylephrine boluses (During)	3.65 ± 0.53	0.42 ± 0.19	0.32 ± 0.09	0.22 ± 0.07	2.61-4.69	0.05-0.79	0.14-0.50	0.08-0.36	0.001
Phenylephrine boluses (After)	1.47 ± 0.36	0.87 ± 0.33	3.60 ± 0.41	4.00 ± 0.49	0.76-2.18	0.22-1.52	2.81-4.39	3.04-4.96	0.001
Total phenylephrine used (µg)	692.33 ± 66.73	1269.33 ± 119.52	591.75 ± 115.25	438.83 ± 82.80	561.64-823.02	1035.97–1502.69	365.90-817.60	276.94-600.72	0.001

Maternal adverse effects

Incidences of nausea, vomiting, dizziness, and related side effects were lowest in Group B (5.0%, 95% CI: 0.0%-10.5%) compared to Groups A (28.3%, 95% CI: 15.5%-37.9%), C (31.7%, 95% CI: 19.9%-43.4%), and D (20.0%, 95% CI: 9.9%-30.1%), showing a statistically significant difference (*P* = 0.016).

Reactive hypertension events occurred most frequently in Group D (38.3%, 95% CI: 25.3%-51.4%) and Group C (33.3%, 95% CI: 20.8%-45.8%), compared to Group A (23.3%, 95% CI: 12.0%-34.7%) and Group B (21.7%, 95% CI: 10.8%-32.6%) (*P* = 0.001).

Bradycardia was markedly more common in Group C (63.3%, 95% CI: 49.4%-73.9%) and Group D (60.0%, 95% CI: 47.6%-72.4%) than in Groups A (26.7%, 95% CI: 15.5%-37.9%) and B (25.0%, 95% CI: 14.0%-35.9%) (*P* = 0.001) (Table 4).

**Table 4:** Maternal adverse effects. CI, confidence interval

Effect	Group A % (95% CI)	Group B % (95% CI)	Group C % (95% CI)	Group D % (95% CI)	*P*-value
Nausea/Vomiting/Dizziness	28.3 (15.5-37.9)	5.0 (0.0-10.5)	31.7 (19.9-43.4)	20.0 (9.9-30.1)	0.016
Reactive hypertension	23.3 (12.0-34.7)	21.7 (10.8-32.6)	33.3 (20.8-45.8)	38.3 (25.3-51.4)	0.001
Bradycardia	26.7 (15.5-37.9)	25.0 (14.0-35.9)	63.3 (49.4-73.9)	60.0 (47.6-72.4)	0.001

At all assessed time points, neonatal APGAR scores were highly favorable across all treatment groups. At 1 minute, the mean APGAR scores ranged from 8.43 (95% CI: 8.23-8.63) in Group D to 8.75 (95% CI: 8.59-8.91) in Group B, with no statistically significant inter-group differences (*P* = 0.726). By 5 minutes, mean APGAR scores increased further, ranging from 9.83 (95% CI: 9.72-9.94) in Group D to 9.97 (95% CI: 9.92-10.02) uniformly in Groups A, B, and C (*P* = 0.115). At 10 minutes, all neonates achieved an APGAR score of 10.00 (95% CI: 10.00-10.00), confirming uniformly excellent neonatal adaptation after delivery, without any significant group differences (*P* = 0.999) (Table 5).​

**Table 5:** APGAR scores. CI, confidence interval

Time point	Group A (mean, 95% CI)	Group B (mean, 95% CI)	Group C (mean, 95% CI)	Group D (mean, 95% CI)	*P*-value
1 minute	8.63 (8.44-8.82)	8.75 (8.59-8.91)	8.50 (8.33-8.67)	8.43 (8.23-8.63)	0.726
5 minutes	9.97 (9.92-10.02)	9.97 (9.92-10.02)	9.97 (9.92-10.02)	9.83 (9.72-9.94)	0.115
10 minutes	10.00 (10.00-10.00)	10.00 (10.00-10.00)	10.00 (10.00-10.00)	10.00 (10.00-10.00)	0.999

All groups demonstrated rapid recovery in APGAR scores after birth, indicating minimal impact of phenylephrine regimen on immediate neonatal well-being.

**Figure 4:**
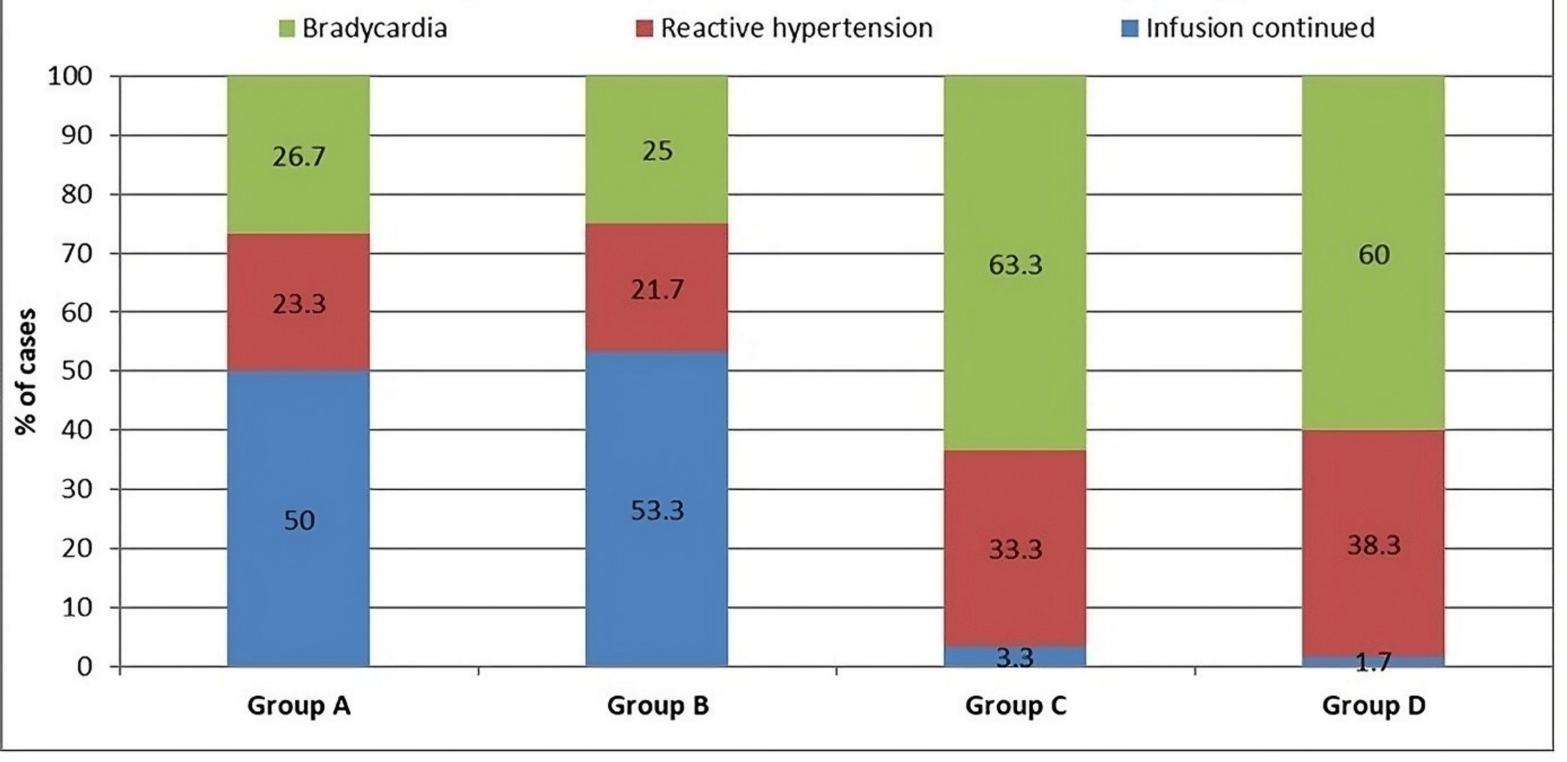
Inter-group comparison of reasons for stopping the infusion. Groups A, B, C, and D indicate phenylephrine infusion rates of 25, 50, 75, and 100 mcg/minute, respectively, administered after spinal anesthesia in elective cesarean section. The figure shows the proportion of patients in each group whose infusion was discontinued due to bradycardia or reactive hypertension. Rates of infusion being discontinued with reason being reactive hypertension were highest in Groups D and C, while infusion being stopped due to bradycardia was also more frequent in Groups C and D. Statistical comparison was performed using chi-square test (*P* < 0.001).

Phenylephrine infusion was continued without any side effect like reflex bradycardia or reactive hypertension in more number of patients in groups A and B as compared to groups C and D. Reactive hypertension was more in the sequence of D > C > A > B. Reflex bradycardia was more in the sequence of C > D > A > B (Figure 4).

The incidence of maternal hypotension varied significantly among groups (*P* = 0.001). Group C exhibited the highest incidence at 25.0% (95% CI: 14.0%-36.0%), followed by Group A at 21.7% (95% CI: 11.3%-32.1%), Group D at 16.7% (95% CI: 7.3%-26.1%), and Group B at the lowest incidence of 1.7% (95% CI: 0.0%-5.0%). These results indicate a statistically significant reduction in hypotension incidence with the phenylephrine infusion rate used in Group B compared to the other regimens (Table 6).

**Table 6:** Incidence of hypotension among different groups. CI, confidence interval

Effect	Group A (%) (95% CI)	Group B (%) (95% CI)	Group C (%) (95% CI)	Group D (%) (95% CI)	*P*-value
Hypotension	21.7 (11.3-32.1)	1.7 (0.0-5.0)	25.0 (14.0-36.0)	16.7 (7.3-26.1)	0.001

In summary, Group B (50 µg/minute phenylephrine infusion) demonstrated the most favorable hemodynamic stability with minimal maternal side effects and comparable neonatal outcomes. The differences in infusion duration, total phenylephrine requirement, and side effect frequency were statistically significant (*P* < 0.05), highlighting 50 µg/minute as the optimal prophylactic dose for maintaining maternal blood pressure during elective cesarean delivery while ensuring neonatal safety.

## Discussion

Spinal, epidural, and general anesthesia are the main modalities used in cesarean section, each offering distinct benefits and drawbacks. General anesthesia is the method of choice in emergent settings such as fetal distress, cord prolapse, antepartum hemorrhage, or placenta previa, because it allows for rapid induction and optimal airway as well as ventilation control, thereby providing more cardiovascular stability and minimizing the risk of hypotension [14]. However, its significant disadvantages include the risk of maternal aspiration, heightened sympathetic stimulation during intubation, risk of intraoperative awareness, and potentially adverse neonatal effects such as flabby baby syndrome due to anesthetic drug transfer, as well as intubation challenges arising from pregnancy-related physiological changes [15].

To mitigate these risks, regional techniques like spinal and epidural anesthesia are often chosen in elective cases. Epidural anesthesia, while generally safer than general anesthesia, has limitations such as delayed onset, patchy or incomplete analgesia, unpredictable block height, and even failure, sometimes even in expert hands. Against this backdrop, spinal anesthesia has emerged as the preferred method for elective cesarean sections. This is due to its speed, reliability, technical ease, profound analgesia, and the added advantage of allowing the mother to remain awake without exposing the fetus to sedatives or depressant drugs [16]. Studies, including those by Das et al. [17] and Allen et al. [13], have noted that spinal anesthesia is associated with decreased operative blood loss and high maternal satisfaction.

However, spinal anesthesia is not without its criticisms, the most significant being the risk of profound hypotension due to sympathetic blockade. This can worsen preexisting supine hypotension and, if unaddressed, leads to reduced uteroplacental perfusion and potentially fetal hypoxemia and acidosis. The importance of proactive prophylaxis rather than reactive management of hypotension has been repeatedly emphasized in the literature (as shown in the work of Cooper et al. [18], Nikooseresht et al. [1], and Das et al. [17]), since prompt prevention supports both maternal and neonatal well-being, reduces maternal nausea and vomiting, and preserves patient confidence in the surgical process.

Given this, the pharmacologic focus has shifted towards the use of vasopressors for maternal hemodynamic support. Historically, both phenylephrine and ephedrine have been used; however, mounting evidence, exemplified by studies from Das et al. [17], Cooper et al. [18], and Nikooseresht et al. [1], indicates that phenylephrine, whether administered as an infusion or a bolus, is superior to ephedrine Notably, phenylephrine results in higher neonatal umbilical cord blood pH, indicating less fetal acidemia and better overall neonatal outcomes. Furthermore, compared to bolus therapy administered after hypotension develops, several studies [19-23] have shown that prophylactic phenylephrine infusions started immediately after spinal block offer superior outcomes, both in terms of maternal blood pressure stability and fetal health.

Our study examined 240 ASA grade II women scheduled for elective cesarean section, all of whom received spinal anesthesia following a 10 mL/kg preload with Ringer’s lactate. We compared the effect of four phenylephrine infusion rates (25, 50, 75, and 100 µg/minute) given at 1 mL/minute to identify the dose most effective in preventing spinal-induced hypotension. Although our sample size was reduced from the planned 300 participants due to the COVID-19 pandemic, our cohort remained larger than those of many comparable studies, including Allen et al. [13], Bauer et al. [24], Kumar et al. [20], and Zinger et al. [25], thereby enhancing the robustness of our findings.

Hypotension was defined using a MAP threshold (≤20% below baseline), whereas most previous studies, including Allen et al. [13], Kee et al. [23], and Das et al. [17], used SBP as their primary parameter. Our rationale was based on the local practice of NIBP monitoring, which reliably measures MAP, and the physiological argument that MAP better reflects both systolic and diastolic pressures, resulting in earlier detection and intervention for hypotension.

Results revealed no significant demographic or intraoperative differences between groups regarding age, weight, comorbidities, or baseline vitals. Block height ranged uniformly from T5 to T6. Most critically, Group B (50 µg/minute) exhibited the lowest incidence of hypotension, both during and after infusion, with minimal reflex bradycardia (25%) and the least nausea/vomiting. Lower doses (25 µg/minute, Group A) were insufficient in hypotension prevention, while higher doses (75 and 100 µg/minute, Groups C and D) often required early stoppage due to hypertension or bradycardia, resulting in a more consistent post-infusion hypotensive episode pattern consistent with Allen et al. [13]. Our protocol was unique in that once an infusion was stopped (either for bradycardia or hypertension), it was not restarted, differing from Allen et al.'s more complex stop/restart approach.

We found that administration of 50 µg/min phenylephrine over a sustained period achieved steady MAP control without frequent adverse effects, despite the group receiving the highest cumulative dose. This supports findings from both Allen et al. [13] and Choudhary et al. [19], who also observed that moderate, continuous infusions outperform either high boluses or rapidly escalating doses.

Neonatal outcomes, as measured by APGAR scores at 1, 5, and 10 minutes, were uniformly excellent (all means within 8.43-8.75 at 1 minute; 9.87-9.97 at 5 minutes; 10 at 10 minutes) across all dosing groups, indicating no detrimental effect even with a larger total phenylephrine dose. This aligns with Das et al. [17], Kee et al. [23], and Siddik-Sayyad et al. [22], who also report good neonatal profiles with prophylactic phenylephrine, although, unlike some, our study did not conduct umbilical cord blood gas analysis due to logistical constraints.

Importantly, while our study supports the benefit of fixed-rate 50 µg/minute phenylephrine infusion for healthy, elective populations, caution is advised in extrapolating these results to high-risk pregnancies, urgent cesarean sections, or women with compromised placental function, as these groups were excluded in this and most contemporary studies like Kee et al. [23] and Allen et al. [13].

Study limitations

Several limitations of this study should be acknowledged. First, the study was conducted at a single center, which may limit the generalizability of findings to different populations and practice settings. Second, while APGAR scores were used to assess neonatal outcomes, more sensitive measures of fetal well-being, such as umbilical cord blood gas analysis, were not included. Third, the study did not investigate the effects of different patient characteristics, such as baseline blood pressure, body mass index, or comorbidities, on the optimal dosing regimen. Additionally, the study protocol used a fixed infusion rate approach, which may not account for individual patient variability in response to spinal anesthesia. Future studies might investigate variable-rate infusion protocols or closed-loop systems that adjust phenylephrine delivery based on real-time hemodynamic parameters.

Future research directions

Future research should focus on several areas to further optimize the use of prophylactic phenylephrine infusion. Investigations into patient-specific factors that might influence optimal dosing, such as baseline cardiovascular status, gestational age, and comorbidities, would help develop more personalized approaches. Additionally, studies comparing prophylactic infusion protocols with reactive management strategies using health economic outcomes would provide valuable information for healthcare decision-making.

The development of closed-loop systems for automated phenylephrine delivery based on continuous hemodynamic monitoring represents an exciting frontier that could further improve the precision and safety of vasopressor administration during cesarean delivery.

## Conclusions

Prophylactic phenylephrine infusion after spinal anesthesia not only reduces the incidence and severity of maternal hypotension during elective caesarean sections but does so without adverse neonatal consequences. A moderate dose of 50 µg/minute provides the best trade-off between efficacy and safety, supporting the current trend in international guidelines.

In conclusion, this study demonstrates that prophylactic phenylephrine infusion at 50 µg/minute represents an optimal strategy for preventing hypotension during elective cesarean delivery. This approach effectively maintains maternal hemodynamic stability, reduces the incidence of hypotension-associated symptoms, minimizes the need for rescue interventions, and maintains excellent neonatal outcomes. The findings support the adoption of this proactive approach in clinical practice as a standard of care for elective cesarean delivery under spinal anesthesia.
